# A review on pathology, mechanism, and therapy for cerebellum and tremor in Parkinson’s disease

**DOI:** 10.1038/s41531-022-00347-2

**Published:** 2022-06-24

**Authors:** Yuke Zhong, Hang Liu, Guohui Liu, Lili Zhao, Chengcheng Dai, Yi Liang, Juncong Du, Xuan Zhou, Lijuan Mo, Changhong Tan, Xinjie Tan, Fen Deng, Xi Liu, Lifen Chen

**Affiliations:** Department of Neurology, The Second Affiliated Hospital of Chongqing Medical University, Chongqing, China

**Keywords:** Parkinson's disease, Parkinson's disease, Neurological manifestations

## Abstract

Tremor is one of the core symptoms of Parkinson’s disease (PD), but its mechanism is poorly understood. The cerebellum is a growing focus in PD-related researches and is reported to play an important role in tremor in PD. The cerebellum may participate in the modulation of tremor amplitude via cerebello-thalamo-cortical circuits. The cerebellar excitatory projections to the ventral intermediate nucleus of the thalamus may be enhanced due to PD-related changes, including dopaminergic/non-dopaminergic system abnormality, white matter damage, and deep nuclei impairment, which may contribute to dysregulation and resistance to levodopa of tremor. This review summarized the pathological, structural, and functional changes of the cerebellum in PD and discussed the role of the cerebellum in PD-related tremor, aiming to provide an overview of the cerebellum-related mechanism of tremor in PD.

## Introduction

Tremor, defined as an involuntary, rhythmic, and oscillatory movement of a body part, is one of the cardinal symptoms of Parkinson’s disease (PD)^1,2^. Traditional taxonomy of PD introduced several subtypes, including tremor-dominant PD and non-tremor-dominant PD (including postural instability and gait disability-dominant PD, akinesia/rigidity-dominant PD)^3^. Recent studies demonstrated there might be different biological bases for different subtypes of PD^4^. Tremor-dominant PD patients tend to have lower proportion of death and disability, slower progression of the disease, better cognitive function, lower burden of nonmotor symptoms, and longer survival time^3,4^. Thus, tremor-dominant PD is considered a benign subtype of PD^5^. However, among all the motor symptoms of PD, the mechanism of tremor is still poorly understood^6^, and its responsiveness to levodopa varies^5,7^.

The role of the cerebellum in the mechanism of tremor in PD has been increasingly focused^8^. The cerebellar output has been verified to modulate tremor-related activity, which arises from globus pallidum and propagates to cerebello-thalamo-cortical (CTC) circuits^9^. Moreover, tremor-related cerebellar activity differs between PD patients with dopamine-responsive tremor and dopamine-resistant tremor^5^, indicating a role of the cerebellum in the responsiveness of tremor to dopamine in PD.

We reviewed the pathological, structural, and functional changes of the cerebellum in PD and discussed the role of the cerebellum in PD-related tremor, aiming to provide an overview of the cerebellum-related mechanism of tremor in PD.

### Pathological changes in the cerebellum in PD

PD is characterized by Lewy body pathology formed by α-synuclein, while cerebellum was thought to be unaffected by Lewy bodies previously^8,10^. However, recent studies discovered α-synuclein-related pathological changes in the cerebellum in PD patients, which may be associated with tremor symptoms. In PD patients, α-synuclein-formed Lewy bodies, which were speculated to originate in the pre-cerebellar brainstem and spread in a prion-like manner, were identified in the cerebellum^11^. Lewy bodies were found mainly in the cerebellar nuclei and adjacent white matters, while cerebellar lobules were only affected mildly^11^. Histologically, in the cerebellum of PD patients, Lewy bodies were found in Bergmann glia in the molecular layer and Purkinje cell axons^12,13^.

PD patients have longer climbing fiber length, more climbing fibers extending into the molecular layer, more climbing fiber-Purkinje cell synapses, and increased percentage of climbing fiber-Purkinje cell synapses on the thin Purkinje cell dendritic branchlets compared with healthy controls, accompanied by torpedoes/swelling of Purkinje cell axons^14,15^. Based on cluster analysis, these pathological changes may form a pattern that predicts the presence of resting tremor, PD patients with lower climbing-fiber synaptic density and a higher Purkinje cell count tend to have rest tremor^15^.

Moreover, iron accumulation has been identified in the deep nuclei of the cerebellum in PD. Using quantitative susceptibility mapping (QSM), iron content in dentate nuclei was found elevated in tremor-dominant PD patients compared with healthy controls and akinesia/rigidity-dominant PD patients, and was proven positively correlated to tremor severity despite subtypes of PD^16–18^. Because iron accumulation may indicate ferroptosis, a nonapoptotic cell death pathway, increased iron content in dentate nuclei suggests a role of dentate nuclei and cerebellum in the pathophysiological mechanism of tremor in PD^16–19^. Notably, ferroptosis may also promote α-synuclein aggregation^16–18^.

Interestingly, both α-synucleinopathies and iron accumulation affect dentate nuclei, which act as the only output nuclei of the cerebellum, indicating a role of the cerebellum in tremor. White matter changes in the cerebellum, especially Purkinje cells and related climbing fiber changes, presented a clinical manifestation-related pattern, suggesting a role of cerebellar network damage in the tremor of PD. Therefore, in PD patients, damage exists in both deep nuclei and white matters of the cerebellum. These pathological changes outline a comprehensive impairment pattern of the cerebellum.

### Tremor-related structural and functional changes in the cerebellum in PD

#### Structural change

In previous studies, several tremor-related structural changes have been identified in the cerebellum of PD. When compared with PD patients without rest tremor, PD patients with rest tremor presented decreased gray matter volume mainly in quadrangular lobe and declive^20^, and tremor-dominant PD patients had decreased gray matter volume in left cerebellar lobule VIIIa compared with akinesia/rigidity-dominant PD patients^21^. Besides, larger volume of cerebellar lobule IV is associated with severer resting tremor in all PD patients^22^. These findings suggest a possible relation between these cerebellar regions and tremor in PD.

Additionally, tremor-dominant PD patients also present decreased gray matter volume in left cerebellar lobule VI, VIIb,VIIIb, and vermal cerebellar lobules VI and VIIIa compared with healthy controls, but such decrease in gray matter volume in these cerebellar regions was not correlated with tremor severity^21^. It has also been reported that no significant difference in gray matter volume and white matter volume exists between PD patients with tremor and healthy controls^23^. The relation of volume changes in these cerebellar regions with tremor needs to be further illustrated.

The findings above present controversial relation between tremor and cerebellar gray matter volume changes in PD. Notably, the atrophy of a lobule may cause the neighboring lobule to be volumetrically larger or vice versa. Therefore, volumetric analysis of cerebellar regions separately may be insufficient for outlining the tremor-related volumetric change of cerebellum in PD. A comprehensive pattern of volumetric change of cerebellum may be helpful for a better understanding of the role of the cerebellum in tremor in PD.

More importantly, although possible relevance exists between volumetric changes and tremor in PD, whether the volumetric change is a causal factor, consequence, or concomitant phenomenon of tremor is unclear. Therefore, in the future, histological research may be necessary for further illustration of the relation between cerebellar change and tremor in PD.

Although there is limited Diffusion tensor imaging (DTI) studies investigating the role of the cerebellum in tremor in PD, a DTI study demonstrated white matter abnormality within multiple tracts including middle cerebellar peduncle and superior cerebellar peduncle compared with healthy controls and non-tremor-dominant PD patients^24^. This study adds a probability of the involvement of the cerebellar white matters in the tremor mechanism in PD. However, another study found no cerebellar white matter change between tremor-dominant PD patients and non-tremor-dominant PD patients^25,26^ (Table 1). Further DTI study investigating the role of the cerebellar white matter changes in the tremor of PD is needed.

#### Functional change

Studies found functional changes in the cerebellum in tremor-dominant PD patients. PD patients presented a tremor-related metabolic pattern in ^18^F-deoxyglucose positron emission tomography (FDG-PET), glucose metabolism in their dentate nuclei and anterior cerebellar lobule (IV and V) was increased in resting state. This increase in glucose metabolism was positively correlated with tremor amplitude and could be suppressed by both VIM and subthalamic nucleus (STN) deep brain stimulation (DBS)^27^.

In resting-state functional MRI (fMRI), tremor-dominant PD patients showed decreased voxel-mirrored homotopic connectivity (VMHC) in the cerebellar posterior lobe. Moreover, VMHC in the cerebellar posterior lobe was reported negatively correlated with tremor severity in PD patients, while the amplitude of low-frequency fluctuations (ALFF) in this region was reported positively correlated with tremor severity^28,29^. Besides, increased local synchronization of activity in cerebellar crus I and cerebellar lobule VI and decreased local synchronization of activity in cerebellar vermis III, cerebellar lobule IV, and cerebellar lobule V in resting-state was also found in tremor-dominant PD patients. However, no correlation between local synchronization of activity in those regions and clinical manifestations was identified^30^. fMRI studies also found changes in functional connectivity between different cerebellar regions. Functional connectivity between cerebellar cortex and dentate nuclei in tremor-dominant PD patients is increased when compared with non-tremor-dominant PD patients^31^. Interestingly, among these functional connectivities, the functional connectivity between dentate nuclei and the cerebellar posterior lobe is positively correlated with tremor severity, while the connectivity between the anterior cerebellar lobules and dentate nuclei presents no correlation with clinical manifestations^31^. Additionally, tremor-dominant PD patients presented decreased connectivity between bilateral cerebellar hemispheres compared with healthy controls^32^.

Functional connectivity between the cerebellum and other structures was also altered. Functional connectivity in tremor-dominant PD patients between cerebellar lobule VI and basal ganglia, between the cerebellum and supplementary motor areas/insula, were found to be increased compared with healthy controls^32^. Connectivity between bilateral dentate nuclei and prefrontal cortex in tremor-dominant PD patients was decreased compared with healthy controls and non-tremor-dominant PD patients^31^. Furthermore, connectivity between these regions was negatively correlated with tremor severity, while connectivity between a region comprising cerebellar lobules V, VI, VII, and VIII, and supplementary motor areas was positively correlated with tremor severity in all PD patients^31,32^ (Table 2).

According to these previous reports, it seems that dentate nuclei plays a key role in cerebellum-related tremor regulation in tremor-dominant PD patients. It is possible that the activation of dentate nuclei or increase of dentate nuclei-cerebellar cortex interaction may enhance tremor, while the prefrontal cortex may suppress tremor via regulating the activity of dentate nuclei. These findings suggest that direct or indirect intervention on dentate nuclei may be a potential target of alleviating tremor in PD. As dentate nuclei lies deep in the cerebellum, it is inconvenient for intervention therapy such as DBS. However, the prefrontal cortex may be a potential target to regulate dentate nuclei more accessibly. Moreover, cerebellar lobules may be involved in the mechanism of tremor in PD via its influence on dentate nuclei activity. The interaction between the bilateral cerebellar hemisphere, cerebellar lobules, and dentate nuclei may also play a role in tremor activity, but the definite mechanism requires further study.

### The role of the cerebellum in tremor in PD

#### Cerebellum may participate in tremor mechanism via cerebello-thalamo-cortical circuit

The basic of recent research on rest tremor and its responsiveness to dopaminergic treatment is the “dimmer switch model”, which is described in a systematic/circuit-level, and this model is also a foundation for the role of the cerebellum in tremor in PD^1^. This model focus on activity within two important tremor-related circuits, the basal ganglia circuits and the cerebello-thalamo-cortical circuits, and the interaction between these two important circuits. According to the “dimmer-switch model”, transient tremor-related activity first arises in the basal ganglia and, more precisely, internal pallidal globus (GPi), possibly as a consequence of pathological activity due to dopamine depletion in striato-pallidal circuit^1,9,33^. This tremor-related activity propagates to cerebello-thalamo-cortical (CTC) circuits via the connection between GPi and motor cortex^5,9,33–35^. Then, tremor-related activity may propagate to the thalamus and cerebellum via cortico-thalamic and cortico-cerebellar connectivity, within the CTC circuits^9,36,37^. And tremor-related activity may continue to exist in the CTC circuits and thus maintain the tremor until another signal that interacts with tremor-related firing in the CTC circuits is generated^1^.

Moreover, cortico-thalamic excitatory projections from the motor cortex to VIM may lead to low-frequency oscillations within the thalamocortical network^37^. On the other hand, the cerebellum influences the thalamus via glutamatergic excitatory projections from cerebellar deep nuclei to VIM^38–41^.

**Fig. 1:**
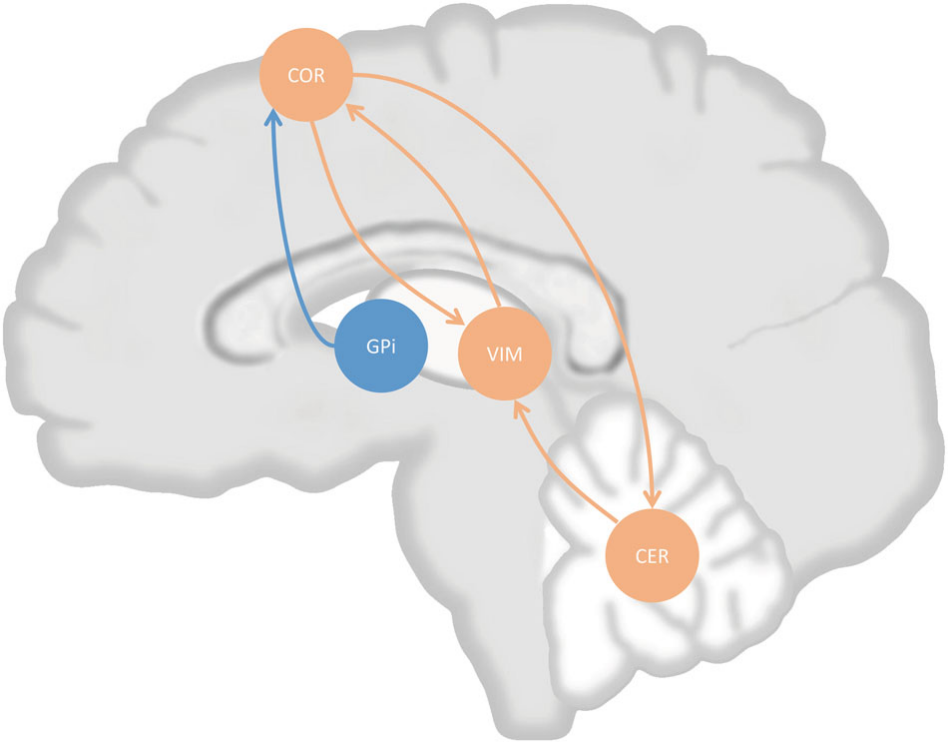
Dimmer switch model of tremor in Parkinson’s disease (PD). Tremor-related activity originates at internal pallidal globus (GPi), which propagate to cortex. Cortex and ventral intermediate nucleus of thalamus (VIM) form a circuit, which is possible the base of tremor-related oscillation. Cerebellum also projects to VIM, this projection possibly modulate amplitude of tremor, while cerebellum was modulated by cerebral cortex. COR cortex, CER cerebellum. Orange arrows indicate projections within cerebello-thalamo-cortical circuit, blue arrow indicates projection from GPi to cortex.

Conclusionally, tremor-related activity origins at GPi and propagates to the motor cortex, where the CTC circuit is activated. Activation of CTC circuits forms cortex-thalamus oscillations, which act as the base of tremor in PD, while the cerebellum modulates tremor amplitude by modulating the activity of VIM in the thalamus. Moreover, the modulation of the cerebellum on VIM is regulated by the motor cortex^9,33^ (Fig. 1).

Before the “dimmer-switch model” was proposed, some previous studies focus on single oscillator (or pacemaker) in tremor mechanism, and localized tremor pacemaker in the basal ganglia or the thalamus according to the ability to oscillate at the same or double frequency of tremor in PD^1^. However, studies found multiple nodes, such as VIM, subthalamic nucleus, and pallidum might serve as a pacemaker, while the modulation of tremor frequency and tremor amplitude seemed independent^9^. Therefore, the “dimmer-switch model” was proposed for a better explanation of the mechanism of tremor in PD.

The “dimmer-switch model” attributes different contributions to different networks (or network nodes), and provide us with a comprehensive view of the tremor mechanism in PD. However, due to the spatial resolution limit of the methods used in current studies, some small nucleus that has been recognized to play a role in tremor mechanism, such as the subthalamic nucleus, is not involved in this model. The absence of these small but important nuclei can make the model incomplete and may lead to errors, especially when speculating the pathways in which tremor-related activity propagates through the brain using dynamic causal modeling (DCM) approaches that require a priori model^1,9,34^. Therefore, it is necessary to be cautious when making anatomical-level speculations on activity propagating pathways based on the “dimmer-switch model”.

In the dimmer switch model, the cerebellum may act as a modulator of tremor-related activity in PD mainly based on the following evidence: (1) there is bidirectional connectivity between the motor cortex and thalamus, forming a circuit that could maintain the tremor-related oscillation independently; (2) cerebellum participates in tremor-related circuit with a unidirectional manner, more specifically cortico→cerebello→thalamic connectivity^9^; (3) cerebellar stimulation could not reset tremor^1^ (Fig. 1).

Additionally, previous studies found that the cerebellum may be involved in processing tremor-related afferents from periphery^42^, while the cortex drives limb tremor^43^, and VIM is involved in both processing tremor-related afferents and driving of limb tremor^44^. Therefore, the cerebellum may be a key structure in the feedback of tremor. Thus, impaired cerebellar function due to PD may contribute to the tremor mechanism in PD via modulating tremor amplitude and feedback of peripheral tremor-related afferents.

Moreover, recent studies found there were bidirectional anatomical connectivities between the cerebellum and basal ganglia, that was: (1) cerebellum nuclei (dentate nuclei) → thalamus → striatum (2) subthalamic nuclei→ pontine nuclei→ cerebellum cortex^45–47^, but whether this bidirectional connectivity has a role in the mechanism of tremor in PD requires further investigation.

#### Neurotransmitters, cerebellum, and tremor in PD

Dopaminergic dysfunction is traditionally regarded as the cause of PD^48^. More specifically, there may be a pallidal and thalamic dopamine depletion in PD patients, subsequent to dopaminergic degeneration in substantia nigra, ventral tegmental area, and mesencephalic retrorubral area^1,49^. According to the “dimmer switch model”, dopamine depletion in pallidum may be the trigger of tremor-related activity in PD^33^. Dopamine depletion may also cause thalamic excitation, which contributes to tremor-related circuit activity^34^. Additionally, although the cerebellum is traditionally regarded as a non-dopaminergic brain area_,_ recent studies demonstrated dopaminergic neurotransmission in the cerebellum^50^. These projections may arise from basal ganglia based on the anatomical connectivity between the cerebellum and basal ganglia^45–47^ and may be influenced by PD-related pathology in the cerebellum^50^. Unfortunately, few studies directly investigated the relevance of the cerebellar dopaminergic system to tremor in PD.

Although dopaminergic dysfunction may contribute to tremor, it seems not the unique cause of tremor in PD. Levodopa treatment presents various effects on tremor in PD patients. In some PD patients, levodopa may present a poor effect on tremor, even though their other symptoms, including bradykinesia and rigidity, are alleviated^5,7,51,52^. This phenomenon persists even at a high dose of levodopa treatment^5,7^, and this tremor is known as dopamine-resistant tremor, which we discussed in the section “Dopamine-resistant Parkinson’s tremor and its relation with cerebellum” below.

Serotonergic neurons in raphe nuclei act as the main source of serotonin in the brain, they gradually degenerate in PD patients as PD pathology progresses^53–55^, leading to serotonin depletion in structures that receive serotonergic projections, such as cortex, thalamus, and basal ganglia^56–59^.

Serotonin system damage plays an important role in the mechanism of tremor in PD. Polymorphism of the SLC6A4 gene encoding the serotonin reuptake transporter is associated with rest tremor in PD^60^. The serotonin-transporter availability in raphe nuclei is decreased in both early stage and advanced tremor-dominant PD patients compared with akinetic-rigidity-dominant PD patients. Moreover, this availability was negatively correlated with tremor severity in all subtypes of PD patients^57,61^. Importantly, serotonergic system degeneration contributes more to tremor than striatal dopaminergic degeneration, and its severity is negatively correlated with responsiveness to levodopa^61^.

Serotonergic neurons influence the cerebellum directly by projection to cerebellar cortex^62–65^ and indirectly via multiple structures^45–47^. Serotonergic neurons in raphe nuclei project to basal ganglia^66^, which connects with the cerebellum bidirectionally^45–47^. Besides, serotonergic neurons also project to structures such as the cortex, several brainstem nuclei, and spinal cord that may influence cerebellar activity^59,67^. Thus, serotonergic system impairment may result in abnormal input into the cerebellum and, consequently, affect tremor-related circuits^1^.

Although a few single photon emission computed tomography (SPECT) studies investigate the state and role of the serotoninergic system in the tremor of PD^57,61^, they did not investigate the role of the serotoninergic system in the cerebellum, possibly due to that ^123^I-FP-CIT in SPECT could act as a tracer for serotonin only in serotonin-transporter-rich region such as raphe nuclei, and cerebellum may not meet the requirement^68^. Besides, in PET and SPECT studies, the cerebellum is traditionally considered as a region with mainly nonspecific binding and is usually used as a reference region for calculating binding ratio^68–70^. Thus, the status and role of the serotonergic system in the cerebellum in PD may be ignored. Radioligand that could selectively reflect serotonin distribution may help investigate the role of the cerebellar serotonergic system in PD tremor.

Locus coeruleus is affected in the early stage of PD pathology spread, which leads to noradrenergic content loss of up to 70% in the brain^71–76^, resulting in decreased noradrenergic projection to cerebellum^77^, thalamus^78^, and motor cortex^78^ in PD. In other words, all structures involved in CTC circuits suffer the loss of noradrenergic input in PD.

Interestingly, tremor-dominant PD patients present less neuronal loss in locus coeruleus^79^, which may indicate a relatively preserved noradrenergic system. Consistently, activating the noradrenergic system (by acute cognitive stress^72^) in human could enhance the activity of the CTC circuit, exacerbate tremor, and suppress the effect of levodopa on tremor in PD patients^72,80^. Activation of the noradrenergic system stimulates both the bottom-up arousal and top-down cognitive control networks, enhances the thalamic activity and CTC circuit activity^80^. On the other hand, inactivating the noradrenergic system by sleep, β-blockers, or placebo, may ameliorate tremor in PD patients^81–85^. These findings support that the noradrenergic system exacerbates tremor in PD.

Interestingly, noradrenergic neurons in locus coeruleus project directly to cerebellum^71,72,86–94^, and may indirectly influence the cerebellum by affecting the thalamus and cortex. It is possible that the direct and indirect effect of noradrenergic system on the cerebellum may influence its modulation on tremor amplitude in PD^1^. Notably, current studies focused on the short-time effect of noradrenergic system change in PD. However, the noradrenergic system is persistently preserved in tremor-dominant PD patients compared with non-tremor dominant PD patients^79^, suggesting a potential background of the continuous noradrenergic system activation, which may lead to a different cerebellar activity status in tremor-dominant PD patients from in non-tremor dominant PD patients. It is a pity that, to our knowledge, there is an absence of direct measurements of noradrenergic activity in vivo, making it difficult to investigate the relation between cerebellar noradrenergic status and tremor in PD patients. Further study is needed to illustrate the role of the noradrenergic system in cerebellum-related tremor activity.

#### Abnormal output of cerebellum may contribute to tremor in PD, possibly via CTC circuit

**Fig. 2:**
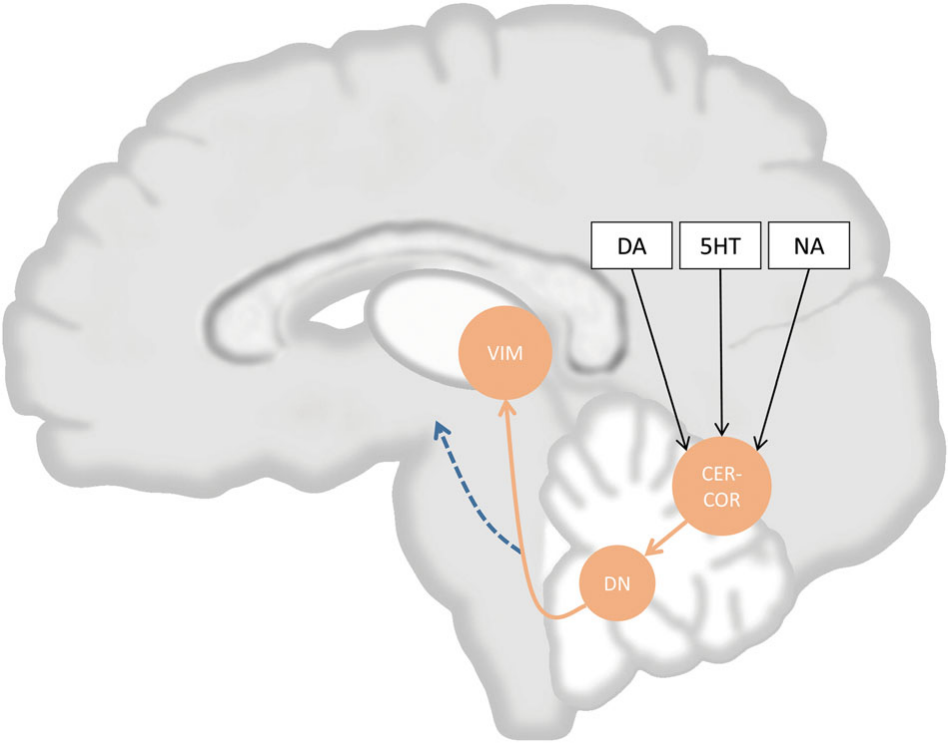
Neurotransmitters modulate tremor activity in Parkinson’s disease (PD) via influencing cerebellar output. Various neurotransmitters including dopamine (DA), serotonin (5HT), and noradrenaline (NA), affect the cerebellar cortex (CER-COR), which modulate the output activity of dentate nuclei (DN). DN project to ventral intermediate nucleus of the thalamus (VIM) and modulate tremor activity. Orange arrows indicate projections between CER-COR, DN, and VIM. The Blue arrows indicate cerebellar output to other brain regions. Black arrows indicate the effect of neurotransmitters.

Cerebellum itself has a complex information processing system. The Glutamatergic granule cell in the cerebellum receives multiple inputs, including dopaminergic input from mesencephalon, serotonergic input from raphe nuclei, and noradrenergic input from locus coeruleus, while all these structures are affected in PD^95–97^ (Fig. 2). For cerebellar projection, GABAergic Purkinje cells form a network in the cerebellum and integrate input from parallel fibers (from granule cell in the cerebellum) and climbing fibers (from inferior olive nuclei), and connect with dentate nuclei^76,95,96^, which project to thalamus and other structures^8,45–47,98,99^. This integrating process could also be damaged by PD-induced Purkinje cell loss, axonal/white matter abnormality, and altered fiber character^11–15^. Moreover, iron accumulation in dentate nuclei in PD patients may also indicate damage of cerebellar output^16–19^, more specifically, the output from cerebellar dentate nuclei to the thalamus via its excitatory glutamatergic projection on VIM^8,45–47,98,99^. Therefore, the external input into the cerebellum, the integrating process in the cerebellum, and the output nuclei are all damaged in PD.

**Fig. 3:**
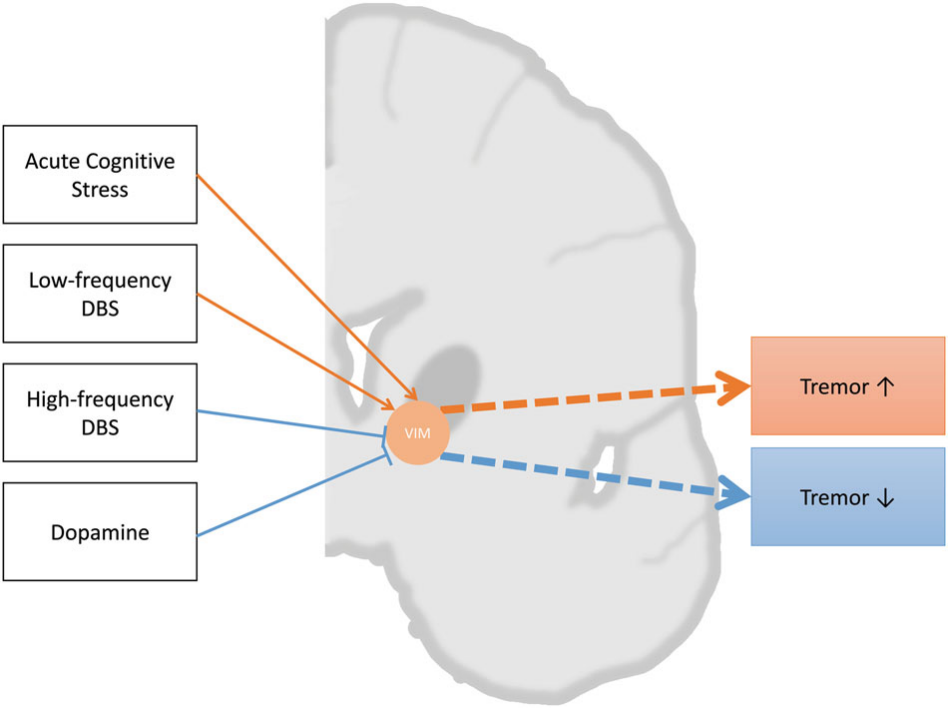
Ventral intermediate nucleus of the thalamus (VIM) plays a central role in tremor activity and may be a target of tremor interference. Acute cognitive stress, deep brain stimulation (DBS), and dopamine could influence VIM and thus regulate tremor. DBS with different frequency present a different effect on tremor activity. Orange arrows indicate excitatory effect, blue arrows indicate inhibitory effect.

VIM receives glutamatergic projections from cortex and cerebellum, and GABAnergic self-inhibitory projection^98^, and it may be a key modulator of tremor in PD patients, according to recent studies reporting the following findings: (1) high-frequency DBS stimulation on VIM may induce synaptic fatigue at excitatory glutamatergic synapses after a transient excitation of these synapses, thereby suppresses tremor in PD patients^8,98^; (2) low-frequency DBS stimulation on VIM that can excite glutamatergic synapses while allowing glutamatergic synaptic vesicle to be replenished worsens tremor in PD patients^98^; (3) excitation of VIM induced by cognitive load was reported to exacerbate tremor in PD patients^80^ (Fig. 3).

Notably, excitatory glutamatergic synapses on VIM are from the cerebellum, at least partially^5,98^, indicating an important role of the cerebellum in the modulation of tremor in PD patients, possibly by its glutamatergic projection. Consistently, levodopa-resistant tremor-dominant PD patients present relatively higher activity in the cerebellum, including dentate nuclei, lobule IV, lobule V, vermis IX, and interposed nuclei^5^. Therefore, the cerebellum may also play an important role in the modulation of tremor in PD via regulating the activity of VIM by its glutamatergic projection. Thus, suppressing cerebellar glutamatergic projection to VIM may be a potential strategy for treating PD tremor.

Additionally, it has been demonstrated in essential tremor that GABAergic projection abruption^15,100–104^, as well as its possible influence on deep nuclei^105,106^, may lead to disinhibition of dentate nuclei, which may serve as the pacemaker and drive the CTC circuit to generate tremor^15,99^. Therefore, it is also possible in PD that abnormal output of dentate nuclei may contribute to disinhibition of the CTC circuit and lead to tremor, but direct evidence is required for this hypothesis.

Conclusionally, in PD, altered input from the dopaminergic, serotonergic and noradrenergic system into the cerebellum, damage of cerebellar integrating process, and dentate nuclei damage may alter cerebellar (excitatory) output at three different levels, and thereby, may possibly influence the modulation of tremor via affecting VIM and then CTC circuit, as indicated by the “dimmer switch model”^1^.

### Cerebellum-related mechanism of therapy for tremor in PD

As the most commonly used drug therapy for PD, levodopa could ameliorate tremor in PD patients by suppressing tremor-onset-related activity in GPi and inhibiting tremor-amplitude-related activity in VIM (where D2 receptor was identified recently)^34^. The latter mechanism possibly decreases/normalizes functional coupling between the thalamus and motor cortical areas^5,107^. The mechanism of VIM inhibiting exists in both dopamine-responsive and dopamine-resistant PD patients, but is more prominent in dopamine-responsive PD patients^5,34^.

Interestingly, levodopa suppresses tremor in PD via inhibiting tremor-amplitude-related activity in VIM, supporting VIM as a key target of levodopa treatment in suppressing tremor in PD^5,34,98,107^. Thereby, abnormally enhanced glutamatergic projection from the cerebellum on VIM may decrease the susceptibility of VIM to dopamine, and thus, results in levodopa resistance^5,34^. Therefore, if further investigated, the cerebellum may be a promising alternative therapeutic target in PD patients with dopamine-resistant tremor.

Dopamine agonists (such as pramipexole)^85,108–110^, monoamine oxidase B (MAOB) inhibitors (such as selegiline, rasagiline)^111–113^, and catechol-O-methyltransferase (COMT) inhibitors (such as entacapone, tolcapone)^85,114^ were also reported to alleviate tremor via different mechanisms. It is easy to understand that COMT inhibitors alleviate tremor by promoting the effect of levodopa^85,114^. Pramipexole has been reported to be effective in some tremor resistant to antiparkinsonian drugs other than pramipexole^110^. However, the effect of pramipexole on tremor-related circuits and cerebellum is unclear. Similarly, the effect of MAOB inhibitors on tremor-related circuits and cerebellum is also unclear, and further investigations are needed.

Additionally, as classical anti-tremor drugs, anticholinergic drugs, including benzhexol, could alleviate tremor by modulating the balance between the dopaminergic and cholinergic systems. However, the specific mechanism and its effect on tremor-related circuits and cerebellum are also poorly understood^115–117^.

Notably, dopamine receptors and choline receptors are expressed in the cerebellum, although at relatively low level^50,118^, it is possible that these drugs (except levodopa) may modulate cerebellar activity and thus, alleviate tremor via CTC circuits. However, no existing direct evidence support this hypothesis; further study is needed.

In addition to drug therapy, non-pharmacological treatment is another approach to alleviate tremor, particularly dopamine-resistant tremor, in PD^119^. Thalamotomy is historically an invasive method of relieving drug-resistant tremor in PD^120,121^. The mechanism may be direct damage to the VIM, which plays an essential role in the tremor mechanism^120^. However, thalamotomy is irreversible and uncontrollable, and may bring complications such as paresthesia and gait disturbance^122^. Even with modification of surgical approach, complications related to surgery itself may occur^122,123^.

DBS is the preferred method for relieving PD tremor compared to thalamotomy currently^122^. VIM-DBS effectively alleviates both dopamine-responsive and dopamine-resistant tremor, but has limited effect on other parkinsonian symptoms such as rigidity and bradykinesia^124^. This puts VIM at a disadvantage compared with other targets for DBS, such as the subthalamic nucleus, which could alleviate multiple symptoms and possibly slow the progression of PD^125^. Anyway, the effectiveness of VIM-DBS provides us with better insight into the tremor mechanism in PD. VIM-DBS was reported to ameliorate tremor by inhibiting neuronal firing in the VIM, and this inhibition occurs after transient neuronal firing in the VIM, which leads to transient worsening of tremor, and finally lead to fatigue of VIM excitatory afferents, and inhibition of VIM activity^98^. Indeed, VIM is nucleus that receives glutamatergic excitatory afferents from the cerebellum^38,39^. Thus, VIM-DBS may alleviate tremor by modulating cerebellum-regulated VIM activity, and thus may change the activity in the CTC circuit. However, the specific mechanism that VIM-DBS ameliorate tremor in PD is still unclear and needs further research.

### Dopamine-resistant Parkinson’s tremor and its relation with cerebellum

The effect of dopamine on tremor in PD is variable between individuals. Zach, et al. Categorized PD patients into three clusters (the dopamine-responsive, intermediate, and dopamine-resistant rest tremor) based on the change of tremor amplitude and the change of tremor power after levodopa challenge^7^. The PD patients with dopamine-responsive rest tremor (PD-RP) display a higher disease severity, longer disease duration, and a higher frequency of accompanying dyskinesia when compared with PD patients with intermediate and dopamine-resistant rest tremor^7^. As mentioned above, VIM inhibition is an essential mechanism for dopamine to alleviate tremor^34^, this inhibition is more significant in PD-RP when compared with dopamine-resistant rest tremor (PD-RS)^5,34^. Furthermore, several brain regions in the cortex, thalamus, and cerebellum present different tremor-related activity between PR-RS and PR-RP^5^. Besides, although not statistically significant, the score for rest tremor severity is lower in PD-RS when compared with PD-RP^5,7^. These studies indicate that there may be a different mechanism underlying the two/three phenotypes as they represent different clinical and pathophysiological characters.

A possible explanation for the above differences in responsiveness of tremor to dopamine between PD-RS and PD-RP may be as follows: Because different brain regions may be affected as the PD pathology progresses^11,53–56,73–78^. At different disease stages, brain regions responsible for tremor may be affected by PD pathology to different degrees. In other words, many brain regions may participate in tremor mechanisms, but at a certain disease stage, one of these regions may account most for tremor, which may result in altered responsiveness of rest tremor to dopamine as the disease progresses^7^. As the cerebellum is mainly affected by various non-dopaminergic neurotransmitters rather than dopaminergic neurotransmitters^5^, resistance to dopamine may occur when the cerebellum accounts most for tremor at a certain disease stage. This speculation is supported by the study of Dirkx, et al., which found increased tremor-related activity in cerebellar lobules IV/V/IX and nuclei^5^.

Further longitudinal researches on the change of tremor-related activity in different brain regions (e.g., cerebellum, thalamus, and cortex), tremor responsiveness to dopamine, and tremor-related activity-dopamine responsiveness relationships as disease progressing are needed for a better understanding of the mechanisms of dopamine-resistant rest tremor and the role of the cerebellum in it.

In conclusion, the cerebellum is affected by α-synuclein-formed Lewy bodies and by iron accumulation in PD, which may induce tremor-related white matter alteration and tremor-specific structural and functional changes in the cerebellum. This damage in the cerebellum, together with damage of other structures, including mesencephalon, raphe nuclei, and locus coeruleus, may alter cerebellar (excitatory) output at three different levels (the input, the integrating process, and the output nuclei). The altered output of the cerebellum to VIM could excite the CTC circuit and enhance the tremor amplitude in PD. The dysregulation of cerebellum-modulated VIM activity may also decrease the susceptibility of VIM to levodopa, thus leading to dopamine-resistant tremor. Currently, few studies investigated the role of the cerebellum in PD-related tremor and its therapy, but still indicated an important role of the cerebellum in the mechanism of PD-related tremor. If further researched, the cerebellum may be a promising target of understanding and treatment of tremor in PD.

**Table 1:** Structural imaging studies reporting on tremor and cerebellum in Parkinson’s disease.

Authors	Study design	Main finding
Benninger et al.^20^	Comparison of GMV in basal ganglia, thalamus, brainstem and cerebellum in PD patients with rest tremor vs. PD patients without rest tremor	Decreased GMV mainly in quadrangular lobe and declive was found in PD patients with rest tremor
Piccinin et al.^21^	Comparison of cerebellar GMV in HC vs. ARPD vs. TPD	(1) Changes in cerebellar GMV seems driven solely by TPD. (2) Decreased GMV in the left cerebellar lobule VIIIa was found in TPD when compared with ARPD. (3) Decreased GMV in multiple cerebellar lobules was found in TPD patients when compared with HC.
Lopez et al.^22^	(1) Comparison of cerebellar lobule volumes in PD patients vs essential tremor patients (2) Correlation of severity of symptoms and lobule volume in PD patients and ET separately.	In PD patients, lobule volume of cerebellar lobule IV was positively correlated with resting tremor and total tremor severity.
Choi et al.^23^	Comparison of volumes of different brain structures in PD patients with tremor vs. ET vs. healthy controls.	No significant difference in GMV and white matter volume existed between PD patients with tremor and HC.
Luo et al.^24^	Comparison of white matter integrity in TPD^a^ vs. NTPD vs. HC by tract-based spatial statistics.	White matter integrity differences in the white matter tract, including middle cerebellar peduncle and superior cerebellar peduncle, existed when compared TPD with HC or NTPD.

*ARPD* akinetic/rigidity-predominant PD patients, *GMV* gray matter volume, *HC* healthy controls, *TPD* tremor-dominant PD patients.

^a^TPD in this study is defined by the presence of a severe tremor and NTPD is defined by the absence of tremor at rest.

**Table 2:** Functional imaging studies reporting on tremor and cerebellum in Parkinson’s disease.

Authors	Study design	Main findings
Mure et al.^27^	(1) Identify the metabolic network in TPD. (2) Identify the correlation between the metabolic network (above) and clinical manifestations/ characters and interventions directed at tremor.	(1) Tremor-related metabolic pattern was characterized by increases in cerebellum/dentate nucleus, primary motor cortex, and caudate/putamen. (2) VIM and STN DBS lead to reduced expression of the tremor-related metabolic pattern. (3) Pattern expression values correlated with tremor amplitude.
Hu et al.^28^	(1) Comparison of homotopic resting-state functional connectivity patterns (revealed by VMHC) in akinetic-rigid PD (ARPD) vs. tremor-dominant PD (TPD) vs. healthy controls (2) Identify the correlation between VMHC values and clinical characters.	(1) TPD exhibited lower VMHC in the posterior lobe of the cerebellum when compared with ARPD and HC. (2) Tremor scores are negatively correlated with VMHC in the posterior lobe of the cerebellum (only) in TPD.
Chen et al.^29^	(1) Comparison of spontaneous neural activity (revealed by ALFF) in resting-state in PIGD vs. TPD vs. HC. (2) Identify the correlation between ALFF values and clinical characters.	(1) TPD exhibited higher ALFF in the cerebellar posterior lobe when compared with PIGD and HC. (2) Tremor scores are positively correlated with ALFF in the cerebellar posterior lobe in all PD patients.
Ma et al.^31^	Comparison of functional connectivity of DN (with other brain structures) in TD vs. PIGD. Identify the correlation between functional connectivity of DN and the tremor severity	(1) In TPD, DN exhibited higher connectivity with the cerebellar anterior lobe and lower connectivity with the prefrontal cortex when compared with HC and PIGD. (2) In TPD, DN exhibited higher connectivity with the cerebellar posterior lobe when compared with PIGD. (3) Connectivity between DN and cerebellar posterior lobe correlated with tremor positively in all PD patients. (4) Connectivity between DN and prefrontal cortex correlated with tremor severity negatively in all PD patients.
Hou et al.^32^	(1) Comparison of functional connectivity (focusing on the basal ganglia (BG) and cerebellum) in TPD vs. PIGD vs. HC (2) Identify the correlation between functional connectivity and tremor severity.	(1) Higher functional connectivity between the cerebellum and paracentral lobule, sensorimotor areas was identified when compared PIGD or TPD with healthy controls. (2) Higher functional connectivity between the BG and cerebellar lobule VI, between the cerebellum and supplementary motor areas (SMA)/insula and lower FC within the cerebellum circuit was found when compared TPD with HC. (3) In all PD patients, functional connectivity between a region comprising cerebellar lobules V, VI, VII, and VIII and supplementary motor areas positively correlated with tremor scores in all PD patients.

*ARPD* akinetic/rigidity-predominant PD patients, *HC* healthy controls, *TPD* tremor-dominant PD patients, *PIGD* postural instability and gait difficulty PD patients, *VIM* ventral intermediate nucleus of the thalamus, *STN* subthalamic nucleus of the thalamus, *DBS* deep brain stimulation, *VMHC* voxel-mirrored homotopic connectivity, *ALFF* increased amplitude of low-frequency fluctuations, *DN* dentate nucleus of the cerebellum.

## Data Availability

Data sharing is not applicable to this article as no datasets were generated or analyzed during the current study.
